# Anterior Interhemispheric Approach for the Surgical Treatment of Azygos Anterior Cerebral Artery Aneurysms: A Case Series

**DOI:** 10.7759/cureus.58808

**Published:** 2024-04-23

**Authors:** Pedro A González Zavala, Jesús E Falcón Molina, Isauro Lozano Guzmán, Miguel A Abdo Toro, Iván Téllez Medina, Rabindranath García López, Zita E Salazar Ramírez, Christian J Sandoval Ramírez

**Affiliations:** 1 Department of Neurosurgery, Hospital de Especialidades, Centro Médico Nacional Siglo XXI, Instituto Mexicano del Seguro Social, Mexico City, MEX; 2 Department of Neurosurgery, Centro Neurológico ABC, Centro Médico American British Cowdray (ABC), Mexico City, MEX

**Keywords:** cerebral aneurysm surgery, anterior cerebral artery, anterior interhemispheric approach, surgical clipping, azygos anterior cerebral artery

## Abstract

The azygos artery is an uncommon vascular variant of the anterior cerebral artery (ACA). This anomaly is associated in a high percentage with aneurysms. Management of azygos ACA aneurysms represents a surgical challenge. We present five patients who underwent microsurgical treatment for distal azygos ACA aneurysms with complex morphology. Four patients showed subarachnoid hemorrhage (SAH) and one complained of sentinel headache. Early preoperative digital subtraction angiography (DSA) or computerized tomography angiography (CTA) was performed. All patients were treated by surgical clipping via an anterior interhemispheric approach. During follow-up, all patients had a satisfactory outcome, with postoperative angiograms showing complete resolution of aneurysms.

## Introduction

The azygos anterior cerebral artery (ACA) is a vascular variant characterized by the formation of a single A2 segment, and its branches supply both hemispheres [1]. The frequency of the azygos ACA variant is reported to be 0-10% [2]. In the literature, the azygos ACA is known by different names, such as arteria termatica, azygos pericallosal artery, unpaired pericallosal artery, unpaired cerebral artery, and common arterial cerebral trunk [3]. 

The presence of the azygos ACA variant is associated with aneurysms in 9-71%. In most cases, they are located at the distal end of the azygos artery, where it branches into the callosomarginal and pericallosal arteries, suggesting a role for hemodynamic stress [3,4]. Most of the azygos ACA aneurysms reported are saccular, but the non-saccular shape could be encountered, which could make them unsuitable for endovascular treatment [3]. Microsurgical treatment via interhemispheric approach allows satisfactory proximal control and safe clip application [4]. This study aims to describe the technical and anatomical aspects of the neurosurgical treatment of azygos ACA aneurysms.

## Case presentation

Between January 2021 and February 2023, 253 patients with aneurysms received surgical treatment in our institution. Azygos ACA aneurysms represented 1.9% of our population studied (Table 1). 

**Table 1:** Demographic and radiological data of the study group. F: female, M: male, FOUR: Full Outline of UnResponsiveness, WFNS: World Federation of Neurosurgical Societies, mRS: modified Rankin Scale

Case no.	Gender	Age	Initial FOUR score	Fisher grade	WFNS grade	Aneurysm morphology	Dome size (mm)	Neck size (mm)	Clipping technique	Outcome mRS/ follow-up (months)
1	F	66	16	0	I	Trilobulated	5 x 9	3.5	Intersecting clipping	0/12
2	M	40	14	3	II	Multilobulated	6 x 11	2.5	Intersecting clipping	0/12
3	F	47	14	4	II	Trilobulated	5 x 8	3	Stacked clipping	2/12
4	F	57	16	3	I	Saccular	4 x 5	3	Stacked clipping	0/4
5	F	56	9	3	V	Multilobulated	6.1 x 7	3.7	Simple clipping	1/24

Case 1

**Figure 1:**
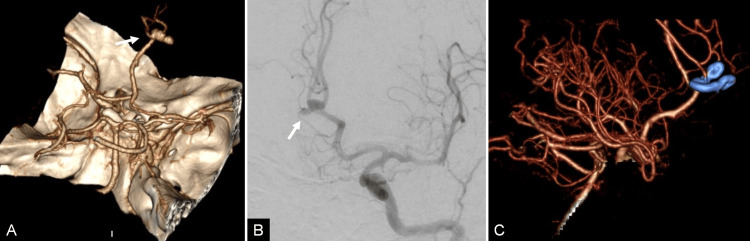
Pre- and postoperative radiological studies of case 1 (A) Preoperative computed tomography angiography (CTA) with 3D reconstruction and (B) left carotid angiogram demonstrating a trilobulated aneurysm (arrow) arising in the azygos bifurcation. (C) Postoperative CTA with 3D reconstruction showing complete obliteration of the aneurysm.

A 66-year-old female with a history of hypertension presented to the emergency department with an intermittent headache, which had increased in frequency and intensity over the last 48 hours. During initial exploration, she had a Full Outline of UnResponsiveness (FOUR) score of 16, without neurological focalization. The results of the serum analysis were normal. A computerized tomography (CT) scan showed no evidence of subarachnoid hemorrhage (SAH). CT angiography (CTA) and digital subtraction angiography (DSA) demonstrated a trilobulated aneurysm measuring approximately 5 mm in width and 9 mm in height, with the neck measuring 3.5 mm at the bifurcation of the azygos ACA (Figures 1A, 1B).

Bicoronal incision, bifrontal craniotomy, and interhemispheric approach were used to clip the aneurysm with an intersecting clip technique. Proximal azygos ACA control was necessary. The control CTA demonstrated aneurysm exclusion and patency of the adjacent vessels (Figure 1C). The patient's postoperative course was uncomplicated, and the patient was discharged six days after admission without neurological deficits. At the one-year follow-up, the patient remained neurologically intact.

Case 2

**Figure 2:**
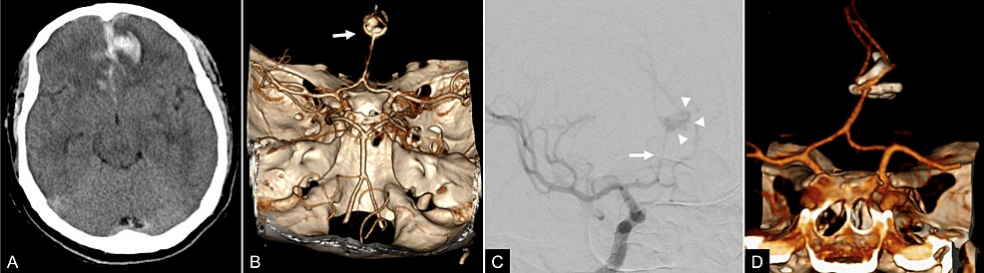
Pre- and postoperative radiological studies of case 2. (A) Computed tomography (CT) sagittal section showing a hyperdense lesion in the anterior interhemispheric fissure (AIHF) associated with subarachnoid hemorrhage. (B) Preoperative CTA with 3D reconstruction and (C) right carotid angiogram scans reveal a single A2 trunk and a multilobulated aneurysm (arrowheads) associated with important vasospasm (arrow). (D) Postoperative CTA with 3D reconstruction demonstrating the disposition of clips applied and resolution of the aneurysm.

A 40-year-old male with no relevant past medical history had a sudden onset of a severe headache, nausea, and vomiting followed by generalized tonic-clonic seizures. Upon admission, he was found drowsy, with neck stiffness. A CT showed a dense clot in the anterior interhemispheric fissure (AIHF) and SAH (Figure 2A). Selective four-vessel cerebral angiography and CTA demonstrated a multilobulated aneurysm at the bifurcation of an azygos ACA, measuring 6 x 11 mm with a neck of 2.5 mm (Figures 2B, 2C).

The surgery was performed using a bifrontal craniotomy and interhemispheric approach. During dissection, there was an incidental aneurysmal rupture, which was controlled with transient dome clipping. Proximal control with a temporary clip for eight minutes was necessary, followed by permanent clipping via the intersecting clip technique. Intraoperative Doppler ultrasonography demonstrated preservation of flow in the azygos ACA and adjacent branches. He stayed in the intensive care unit (ICU) under invasive mechanical ventilation and was extubated four days after surgery. He required antibiotic treatment for pneumonia. Postoperative CTA demonstrated complete aneurysm occlusion (Figure 2D). The patient was discharged nine days after admission, with a FOUR score of 16. After one year of follow-up, he had a good recovery without a neurological deficit.

Case 3

**Figure 3:**
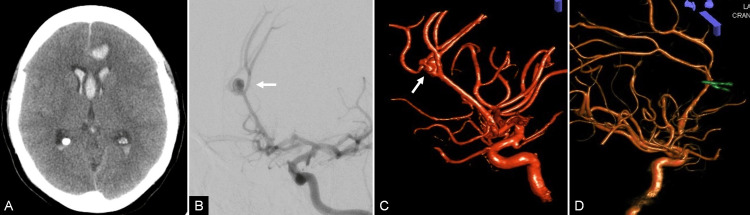
Pre- and postoperative radiological studies of case 3. (A) CT axial section reveals a hyperdense image in the AIHF, a hemorrhage in the genu of the corpus callosum, and cerebral edema. (B) Preoperative carotid angiography with (C) 3D reconstruction showing a distal multilobed azygos anterior cerebral artery (ACA) aneurysm (arrow) with superior projection. (D) Postoperative angiogram with 3D reconstruction depicting total exclusion of the aneurysm. Flow in the callosomarginal arteries was preserved.

A 47-year-old female with a prior medical history of untreated hypertension presented to the emergency department with severe headache, diaphoresis, and somnolence. Upon initial neurological examination, she showed drowsy mental status and dysarthria. A CT scan revealed SAH within the AIHF and blood in the lateral ventricles (Figure 3A). DSA was performed, identifying a trilobulated aneurysm from the azygos ACA bifurcation, measuring 5 x 8 mm with a neck of 3 mm (Figure 3B, 3C).

The patient underwent a right frontal craniotomy and an interhemispheric approach. Temporary clipping was performed to dissect and subsequently exclude the aneurysm with the stacked clipping technique. She required a tracheostomy, antibiotic treatment for pneumonia, and a long stay in the ICU. Postoperative control angiography showed complete obliteration of the aneurysm and preservation of collateral circulation (Figure 3D). She was discharged home 20 days after admission with a FOUR score of 15. One year after surgery, her FOUR score was 16 with only mild left hemiparesis revealed at exploration.

Case 4

**Figure 4:**
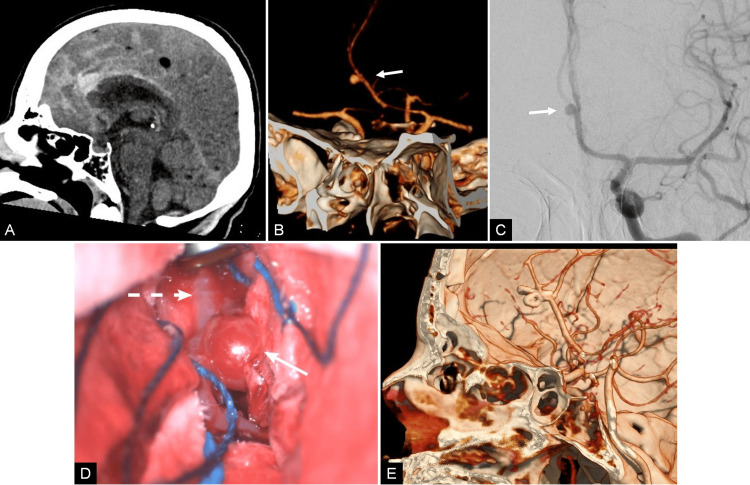
Pre- and postoperative radiological studies of case 4. (A) CT sagittal section showing diffuse hyperdense images in the pericallosal cistern and interhemispheric fissure. (B) Preoperative CTA with 3D reconstruction and (C) left carotid angiography in anteroposterior projection showing a saccular distal azygos aneurysm (arrow). (D) Surgical view depicting temporary clipping of the azygos ACA (dashed arrow) and dissection of the AIHF depicting a distal saccular aneurysm (arrow). (E) Postoperative CTA with 3D reconstruction demonstrates the exclusion of the aneurysm.

A 57-year-old female was admitted for a worsening headache and vomiting for the last four days. She had a history of controlled diabetes and hypertension. The neurological exam was normal, with a FOUR score of 16. A CT scan revealed SAH and a dense clot in the AIHF (Figure 4A). CTA and DSA revealed a saccular aneurysm measuring 4 x 5 mm with a neck of 3 mm, located on the distal part of the azygos trunk (Figures 4B, 4C).

The aneurysm was clipped with the stacked clipping technique with prior azygos proximal control via a bifrontal craniotomy and an interhemispheric approach (Figure 4D). She required a prolonged stay in the ICU, but she did not need a tracheostomy. Postoperative control CTA proved total obliteration of the aneurysm (Figure 3E). The neurological status of the patient improved gradually, and the patient was discharged after 13 days of hospitalization. In the four months following consultation, she was in excellent condition and did not develop any neurological deficits. 

Case 5

**Figure 5:**
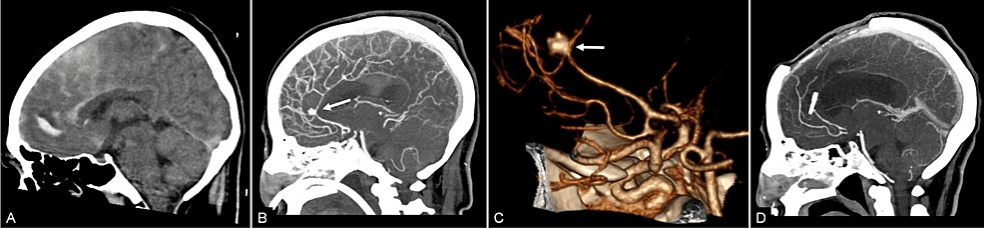
Pre- and postoperative radiological studies of case 5. (A) CT sagittal section showing hyperdense images in the medial surface of frontal lobes and pericallosal cistern. (B) Preoperative CTA sagittal view and (C) 3D reconstruction demonstrating a multilobulated azygos aneurysm (arrow). (D) Postoperative CTA sagittal section reveals frontal craniectomy and exclusion of the aneurysm.

A 56-year-old female with a prior medical history of hypertension was found at home unconscious. Upon admission, neurological examination found a patient with a FOUR score of 9, and intubation was deemed necessary. An urgent CT scan was performed and showed a thick clot in the AIHF (Figure 5A). There was no hydrocephalus. CTA was performed and revealed a multilobulated aneurysm on the distal portion of the azygos ACA, measuring 6.1 x 7 mm with a neck of 3.7 mm (Figure 5B, 5C).

The aneurysm was clipped through a bifrontal craniotomy and interhemispheric approach. Aneurysm was dissected and clipped with a simple clipping technique. The bone flap was not replaced due to the evidence of cerebral edema. She required a prolonged stay at the UCI for two weeks, and she subsequently underwent tracheostomy and gastrostomy. A postoperative CTA scan revealed that the aneurysm was occluded (Figure 5D). The patient was discharged home on the seventeenth postoperative day with a FOUR score of 15. The patient’s neurological status continued to improve. Two years after surgery, she complained of a mild memory deficit.

## Discussion

Anatomical anomalies of the ACA complex have been reported in 23% of cases [5]. Baptista classified the variation of ACA A2 into three types: type 1, azygos ACA; type 2, bihemispheric ACA; and type 3, accessory ACA. Type 1 variation is the less frequent anomaly described [6].

The development of the azygos ACA is due to the abnormal fusion of the paired A2 segment from the medial branch of the primitive olfactory artery at the 16-18 mm stage of embryogenesis. Another theory is the persistence of the median artery of the corpus callosum at the 20-24 mm stage with regression or lack of development of the ACA [7,8].

Azygos ACA is associated with other embryonal anomalies, such as agenesis of the corpus callosum, lipoma, prosencephaly, arteriovenous malformation, and porencephalic cysts [4]. None of these entities were found in our series. Unilateral vertebral artery hypoplasia and ACA A1 segment hypoplasia are vascular variants accompanying azygos ACA [3]. In one patient, we found A1 hypoplasia.

The azygos ACA bifurcates into bilateral pericallosal and callosomarginal arteries supplying the medial surface of both frontal lobes and a large part of the corpus callosum [8]. Beyhan et al., after a review of 57 cases of azygos ACA, proposed a classification of anatomical branching levels divided into four types: type A, branching at the root of A2; type B, branching at the corpus callosum; type C, branching between the corpus callosum genu and the body middle; and type D, branching after the body middle level of the corpus callosum [3]. In all the cases we reported, the branching level of the azygos ACA was type C.

A single A2 segment is associated with cerebral aneurysms. The majority of azygos aneurysms are located at the distal portion of the artery where it bifurcates. Kaspera et al. reported no difference between blood flow in the azygos ACA and control groups. The complex geometry at the bifurcation of the azygos ACA and an enlarged single vessel with a bend at the genu of the corpus callosum may be risky factors predisposing patients to aneurysm formation [9].

Azygos ACA aneurysms may be asymptomatic or symptomatic, similar to distal anterior cerebral artery (DACA) aneurysms. The main symptoms caused by rupture are headache, seizures, nausea, vomiting, loss of consciousness, and, particularly, cognitive deficits. The rupture of DACA aneurysms causes parenchymal hemorrhages located in the frontal lobes, corpus callosum, and cingulate gyrus [4,7,8,10]. 

Treatment of DACA aneurysms is performed by surgical clipping or endovascular coiling, with high rates of technical success of over 90% and similar neurological morbidity/mortality rates. The surgical modality is associated with a superior complete occlusion rate (94% vs. 62%), lower rates of aneurysm recurrence (3% vs. 18%), and lower rates of aneurysm rebleeding (2% vs. 4%) [11]. In our center, there are both treatment modalities, but by multidisciplinary consensus, microsurgical treatment was preferred due to the presence of the azygos ACA variant, the architecture of the aneurysms, and the presence in most cases of intraparenchymal hematoma that required drainage.

For approach selection, the azygos branching level, aneurysm morphology, and relation with the corpus callosum were taken into account. In the literature, the most frequently reported morphologies are saccular aneurysms and minor cases with non-saccular or complex (multilobulated) shapes. Cases of non-saccular, broad-based, or multilobulated aneurysms require complex clip application and carry higher risks [12]. We described four cases in which angiography studies exposed a tri- or multilobulated dome.

Aneurysms at this topography represent a surgical challenge. The treatment of aneurysms with complex morphology is suitable by an anterior interhemispheric approach that provides access to deep parafalcine and paraventricular structures through the interhemispheric fissure [13]. We performed a bifrontal craniotomy in most cases and a right frontal craniotomy in one case. Unilateral exposure could be considered when there are no bridging veins that would obstruct a unilateral anterior interhemispheric approach [14]. Ligation and division of the anterior third of the superior sagittal sinus is a measure to provide appropriate exposure with minor complications; however, cerebral edema and venous infarction may occur [15]. In one case, we considered it without further complications. 

The interhemispheric fissure dissection disposes of a natural corridor of the pericallosal artery route; however, in some cases, the cortical surfaces of the medial frontal lobes can be in close relationship or overlapping, making dissection difficult. Furthermore, the pericallosal and interhemispheric cisterns may be lost due to the inflammatory changes of the hemorrhage, which makes it difficult for the surgical field to create a working corridor [16]. The use of neuronavigation combined with CT three-dimensional angiography can improve the accuracy and approach design in intracranial distal aneurysm surgery [17].

In DACA aneurysms and particularly in these atypical lesions, complex vascular structures wrapping around the aneurysm require temporary clipping for neck dissection. Furthermore, during surgery, tentative clipping, multiple clips, trapping, revascularization, and neck reconstruction may be needed [12,18]. Frequently, the corpus callosum overlies the lesion, so the anterior transcallosal approach is an option to achieve proximal vascular control. 

Temporary clipping is an important intraoperative tool that facilitates dissection and provides a view angle of the aneurysm complex prior to definitive clipping. A prolonged duration of temporary clipping of the proximal A2 increases the potential risk of developing medial frontal bilateral infarcts [19]. Only case 5 developed bilateral ACA infarcts and had delayed recovery.

Psychiatric evaluation and support are important because hemorrhagic events in this location can frequently generate psychiatric symptoms due to the involvement of the cingulate gyrus and limbic structures [10,20]. All patients at follow-up had good clinical outcomes with minor sequelae.

## Conclusions

The incidence of the azygos ACA anomaly is relatively uncommon but is highly associated with distal aneurysms. Preoperative CTA and carotid angiography images are imperative for planning surgical intervention. The anterior interhemispheric approach is reliable for the treatment of distal azygos ACA aneurysms. These aneurysms usually have complex morphology and may require temporal clipping and different clipping techniques to secure total exclusion from the circulation. Recognition of anatomic structures is needed to avoid ischemic complications, which are the most commonly associated adverse events.
